# Reply to “Challenges to the Long‐Term Safety Claims of Extremely Low‐Dose Amiodarone: Pulmonary, Thyroid, Hepatic, and Statistical Concerns”

**DOI:** 10.1002/joa3.70194

**Published:** 2025-09-12

**Authors:** Kentaro Yoshida

**Affiliations:** ^1^ Department of Cardiology Institute of Medicine, University of Tsukuba Tsukuba Japan; ^2^ Department of Cardiology Ibaraki Prefectural Central Hospital Kasama Japan

Dear Editor,

We appreciate and thank Rasheed et al. very much for their interest in our article [1] and for highlighting important limitations. However, we believe that these authors did not well understand the most unique point in our study compared with previous studies. First of all, the strength of the present study was its focus on amiodarone 50 mg daily. There are no or few organized data on amiodarone 50 mg therapy in previous studies, as we described in detail in the Discussion section. We would encourage Rasheed et al. to correctly recognize that in most previous studies, “low‐dose” would actually mean 200 mg daily. Although we agree that our study size was not so large, our focus on a 50 mg dose was still a significant strength of the study, and we believe that our organized data on amiodarone 50 mg therapy are quite novel and worthy of publication.

Rasheed et al. likely misunderstood the results of the previous study by Tsaban et al. that was also cited in our manuscript. Although amiodarone did show a small clinically marginal statistical association with increased risk of interstitial lung disease between 2 and 8 years of follow‐up in that study, those authors concluded that this increased risk was clinically negligible and did not significantly affect the patients' overall prognosis because the incidence of interstitial lung disease was low (~2%) and, of note, the mortality risk was lower among patients exposed to amiodarone and remained so for all follow‐up years. We truly sympathize with the final description by Tsaban et al. that “the results of this study may encourage an increase in amiodarone use for rhythm control in AF.” Again, the median daily amiodarone dose was approximately 200 mg in that study, much higher than the dose in our study, making direct comparison with our study difficult.

We agree that thyrotoxicosis is a clinically critical side effect of amiodarone use. However, Guðjónsson et al. never emphasized dose‐independent risks in thyroid dysfunction in their study. On the contrary, a higher dose of amiodarone was actually associated with an increased risk of thyrotoxicosis [HR 2.0 (95% CI 1.1–3.5)]. The mean daily amiodarone dose of 193 mg in their study—again, much higher than that in our study—makes it difficult to directly compare their study with ours.

We agree that hepatotoxicity may be underdiagnosed in amiodarone use because abnormal liver function and symptoms are not necessarily apparent even if hepatotoxicity is present. In our cohort, no patients had abnormal liver tests or suffered from liver dysfunction. Dual‐energy computed tomography may be a useful modality that can contribute to the early detection of hepatotoxicity, but such an assessment was beyond the scope of our retrospective study. Also, the assessment and description of all three of these toxicities (lung, thyroid, and liver) in the manuscript of a study would be difficult due to the word limits imposed by the *Journal of Arrhythmia*. Again, the daily amiodarone dose was 200 mg in all 3 of the patients reported in the case series.

We further appreciate the authors informing us of the interesting study by Kwok et al., which included many more patients (*N* = 1786) taking amiodarone and suggested a prediction score for the development of interstitial pneumonia. We agree that the small number of included patients and lack of multivariable analysis were important limitations of our study. Thus, the facts that the mean daily amiodarone dose was more than 150 mg and the cumulative effect with prolonged exposure was a risk factor also in the Kwok et al. study have encouraged us to conduct a future study with a larger cohort for a more accurate assessment of safety in amiodarone 50 mg daily therapy.

## Disclosure

The author has nothing to report.

## Conflicts of Interest

The author declares no conflicts of interest.
